# Associations Between Halitosis and Craniofacial Morphology, Salivary Biochemical Parameters, and Mouth Breathing in Adult Patients with Malocclusion: A Cross-Sectional Study

**DOI:** 10.3390/jcm14238293

**Published:** 2025-11-22

**Authors:** Koh Kikuchi, Yudai Shimpo, Toshiko Sekiya, Natsuki Shiina, Mami Kiwada, Sakurako Inaba, Yoshiaki Nomura, Hiroshi Tomonari

**Affiliations:** 1 Department of Orthodontics, Tsurumi University School of Dental Medicine, Yokohama 230-8501, Japan; pd23003@stu.tsurumi-u.ac.jp (K.K.); sekiya-t@tsurumi-u.ac.jp (T.S.); piinurts@gmail.com (N.S.); mamygaga116@gmail.com (M.K.); skrk.i3105@gmail.com (S.I.); tomonari-h@tsurumi-u.ac.jp (H.T.); 2 Institute of Photochemistry and Photofunctional Materials, University of Shanghai for Science and Technology, Shanghai 200093, China; nomura-y@sirius.ocn.ne.jp

**Keywords:** halitosis, malocclusion, interincisal angle, salivary ammonia, mouth breathing, volatile sulfur compounds, orthodontics

## Abstract

**Background/Objectives:** Halitosis is a common oral condition primarily caused by volatile sulfur compounds (VSCs) produced in the oral cavity. Although previous studies have suggested that craniofacial morphology, salivary biochemical characteristics, and functional breathing patterns may relate to malodor development, their combined influence in adults with malocclusion has not been fully clarified. This study aimed to investigate the relationships among craniofacial morphology, salivary biochemical parameters, and mouth breathing in adult patients with malocclusion. **Methods:** This retrospective cross-sectional study included 234 adults with malocclusion (75 males and 159 females; mean age 29.0 ± 9.5 years). Halitosis was quantified using gas chromatography, and participants were classified into halitosis-positive (total VSC ≥ 150 ppb, *n* = 79) and halitosis-negative groups (total VSC < 150 ppb, *n* = 155). Craniofacial morphology was evaluated using lateral cephalometric analysis, salivary biochemical factors were assessed using a multi-item saliva testing system and Saxon test, and mouth breathing was assessed based on standardized clinical indicators. Group comparisons, multiple linear regression, and logistic regression analyses were performed. **Results:** The halitosis-positive group demonstrated a larger ANB angle, increased overjet, smaller interincisal angle, and higher salivary ammonia levels compared with the halitosis-negative group (*p* < 0.05). Multiple linear regression identified the interincisal angle as the only independent predictor of total VSC concentration (β = −4.57 per degree reduction in interincisal angle, *p* = 0.019). Logistic regression revealed that mouth breathing significantly increased the likelihood of halitosis (OR = 4.68, 95% CI: 2.62–8.38). **Conclusions:** Craniofacial morphology, salivary biochemical environment, and breathing mode collectively influence halitosis in adults with malocclusion. Incorporating assessment of incisor inclination, salivary ammonia levels, and mouth breathing into orthodontic evaluation may support early identification and preventive management of patients at risk for oral malodor.

## 1. Introduction

Halitosis, commonly known as oral malodor, is a prevalent oral health condition that affects nearly half of the global population and can have profound psychological and social impacts [1,2]. Most cases—approximately 80–90%—originate within the oral cavity and are primarily associated with microbial metabolism of sulfur-containing amino acids, producing volatile sulfur compounds (VSCs) such as hydrogen sulfide (H_2_S), methyl mercaptan (CH_3_SH), and dimethyl sulfide ((CH_3_)_2_S) [3,4]. These volatile compounds are mainly generated on the tongue dorsum, dental plaque, saliva, and periodontal pockets, where anaerobic Gram-negative bacteria proliferate under low-oxygen conditions [5,6,7]. In addition to microbial activity, host-related factors such as salivary flow rate, pH, buffering capacity, and biochemical composition play important modulatory roles in determining oral odor intensity [8,9].

In particular, saliva serves as a critical component of oral homeostasis by lubricating oral tissues, buffering acids, and maintaining microbial equilibrium [8,9]. Decreased salivary secretion or altered composition is associated with oral dryness, impaired clearance of substrates, and increased bacterial activity—all of which can exacerbate halitosis [9,10]. Numerous proteomic and biochemical studies have revealed that salivary proteins such as mucins, statherin, cystatins, and histatins have protective effects by stabilizing enamel minerals and inhibiting bacterial adhesion [11]. Similarly, changes in salivary pH and ammonia concentration can influence the growth of anaerobes and the production of VSCs [12,13]. Diagnostic tools such as a multi-item saliva testing system and the Saxon test provide quantitative evaluation of these salivary parameters and are increasingly used to assess caries and oral environment risk in orthodontic populations [14,15,16].

In addition to biochemical factors, structural and functional aspects of the craniofacial complex may also influence oral malodor. Skeletal discrepancy, particularly skeletal Class II relationships (i.e., a tendency toward maxillary protrusion), has been reported to be associated with a larger ANB angle and a smaller interincisal angle, which reflects more pronounced anterior dentoalveolar inclination [17,18,19]. This morphological imbalance often accompanies mouth breathing, a condition in which air passes through the oral cavity instead of the nasal airway. Mouth breathing has been widely recognized as a functional habit with significant implications for maxillofacial growth, occlusal development, and oral health [20,21]. Pediatric and adolescent studies have consistently demonstrated that chronic mouth breathers exhibit increased lower facial height, clockwise mandibular rotation, and narrowed upper airway dimensions compared with nasal breathers [22,23,24].

Mouth breathing is often first recognized during childhood due to adenoidal hypertrophy or allergic rhinitis, but several reports indicate that it may persist into adulthood even after airway obstruction is resolved, largely due to neuromuscular adaptation [21,22,23,24]. Adults with habitual mouth breathing frequently present with skeletal Class II or vertical growth patterns, lip incompetence, and decreased oral moisture [21,22,23,24].

Malocclusion, particularly dental crowding, has been reported to impair effective oral hygiene by creating areas of plaque retention and food stagnation, thereby promoting tongue coating accumulation and anaerobic bacterial proliferation that contribute to oral malodor [25,26]. Moreover, patients with higher Oral Hygiene Index–Simplified (OHI-S) scores tend to exhibit higher Index of Orthodontic Treatment Need (IOTN) scores, indicating that greater crowding severity is associated with increased difficulty in maintaining oral cleanliness [25]. Several clinical studies have also reported that individuals with untreated malocclusion exhibit higher volatile sulfur compound (VSC) levels and more frequent oral malodor compared with those with normal occlusion [27,28,29].

Furthermore, Zurfluh et al. (2013) demonstrated that when oral hygiene access becomes mechanically compromised, anaerobic bacterial activity and malodor intensify, illustrating how reduced cleansing efficiency contributes to an oral environment favorable for VSC production [30]. Additionally, Grippaudo et al. (2016) highlighted that mouth breathing and oral habits commonly associated with malocclusion further modify salivary dynamics and oral microbiota stability, thereby compounding the risk of VSC accumulation [31]. These findings collectively suggest that malocclusion-related morphological and functional conditions may be associated with increased susceptibility to halitosis even in the absence of orthodontic appliances or active treatment.

Despite these established findings, few studies have examined the combined impact of structural, functional, and biochemical factors on halitosis in adults with malocclusion [29]. Most existing literature has focused either on pediatric populations or on orthodontic treatment effects in isolation. Furthermore, the relationship between cephalometric morphology, salivary parameters, and mouth breathing—three interrelated dimensions of oral function—has not been systematically explored in adults. Understanding this relationship could provide a more comprehensive view of halitosis pathophysiology and help identify risk factors relevant to orthodontic diagnosis and management. To clarify the theoretical basis underlying these associations, we propose a conceptual model illustrating the hypothesized directional pathways linking craniofacial morphology, mouth breathing, salivary conditions, and halitosis (Figure S1).

Therefore, the present study aimed to elucidate the multifactorial relationships among halitosis, craniofacial morphology, salivary biochemical parameters, and mouth breathing in adult patients with malocclusion. Using gas chromatography for VSC quantification, lateral cephalometric analysis, a multi-item saliva testing system, and the Saxon test, this cross-sectional study sought to determine which morphological and physiological variables are most closely related to oral malodor. In addition, binary logistic regression analysis was conducted to estimate odds ratios for the presence of halitosis associated with mouth breathing and other contributing factors, providing a quantitative measure of their relative impact. By integrating structural and functional diagnostic perspectives, this study provides novel insights into how craniofacial features and oral environment collectively contribute to halitosis in adults with malocclusion—an area that remains underexplored in contemporary orthodontic research.

## 2. Materials and Methods

### 2.1. Subjects, Eligibility Criteria and Ethics

This retrospective cross-sectional study included adult patients with malocclusion who visited the Department of Orthodontics at Tsurumi University Dental Hospital between October 2019 and March 2021. A total of 234 patients in the permanent dentition stage (75 males and 159 females; mean age 29.0 ± 9.5 years) were enrolled based on the availability of complete initial diagnostic records.

Inclusion criteria were as follows: (1) permanent dentition, (2) no systemic diseases affecting saliva secretion or nitrogen metabolism, and (3) availability of complete pre-treatment diagnostic records including lateral cephalograms, clinical photographs, dental casts, gas chromatography-based halitosis measurement, a multi-item saliva testing system profile, and the Saxon test results. These criteria were based on previously established clinical assessment protocols for malocclusion and oral function [28,32,33].

Exclusion criteria included:

(1) active dental caries or periodontitis (probing depth ≥ 4 mm or bleeding on probing),
(2) poor oral hygiene (plaque control record ≥ 30%),
(3) smoking habit,
(4) use of systemic antibiotics or antimicrobial oral rinses within the preceding two months, as long-term use of antimicrobial mouth rinses has been shown to alter oral microbiota composition and the biochemical environment of the oral cavity [32,33],
(5) history of psychiatric or neurological disorders,
(6) congenital craniofacial anomalies or multiple tooth agenesis,
(7) history of orthodontic treatment, and
(8) nasal obstruction or allergic rhinitis affecting habitual breathing patterns, meaning participants with active symptoms at the time of examination were excluded to avoid confounding effects of ongoing airway blockage.

All clinical examinations and diagnostic evaluations were performed at the Department of Orthodontics by trained clinicians. This retrospective study was approved by the Research Ethics Committee of Tsurumi University School of Dental Medicine (Approval No. 1728; approved on 29 August 2019). This study was conducted in accordance with the principles of the Declaration of Helsinki. Written informed consent was obtained from all participants prior to participation.

### 2.2. Halitosis Assessment and Group Classification

Halitosis was objectively quantified using gas chromatography (Oral Chroma™ CHM-2, Nissha FIS, Osaka, Japan), which separately measures hydrogen sulfide (H_2_S), methyl mercaptan (CH_3_SH), and dimethyl sulfide ((CH_3_)_2_S). Gas chromatography is recognized as a reliable and reproducible method for evaluating intraoral volatile sulfur compounds (VSCs) in both clinical and research settings [32,33].

To minimize short-term variability in intra-oral gas concentrations, all measurements were standardized as follows: participants were instructed to (1) refrain from eating or drinking except water for at least 2 h, (2) avoid consumption of odor-inducing foods (e.g., garlic, spices) for 24 h, and (3) avoid the use of any mouthwash on the day of measurement in order to prevent transient suppression of VSC production associated with recent rinsing. After maintaining lip closure and nasal breathing for 30 s, an oral gas sample of 1.0 mL was collected using a disposable syringe. The syringe hub was immediately inserted into the injection port of the Oral Chroma device, and 0.5 mL of the sample air was injected for chromatographic analysis according to the manufacturer’s protocol [32,33].

For quantitative evaluation, the total VSC concentration was defined as the sum of H_2_S, CH_3_SH, and (CH_3_)_2_S. A total VSC level of ≥150 ppb was used as the threshold for clinically perceptible halitosis, based on organoleptic recognition thresholds established in previous halitosis research [34,35]. Accordingly, participants were categorized into the halitosis-positive group (total VSC ≥ 150 ppb, *n* = 79) and the halitosis-negative group (total VSC < 150 ppb, *n* = 155). These group classifications were used for subsequent statistical comparisons and multivariable analyses.

### 2.3. Evaluation of Craniofacial Morphology

**Figure 1:**
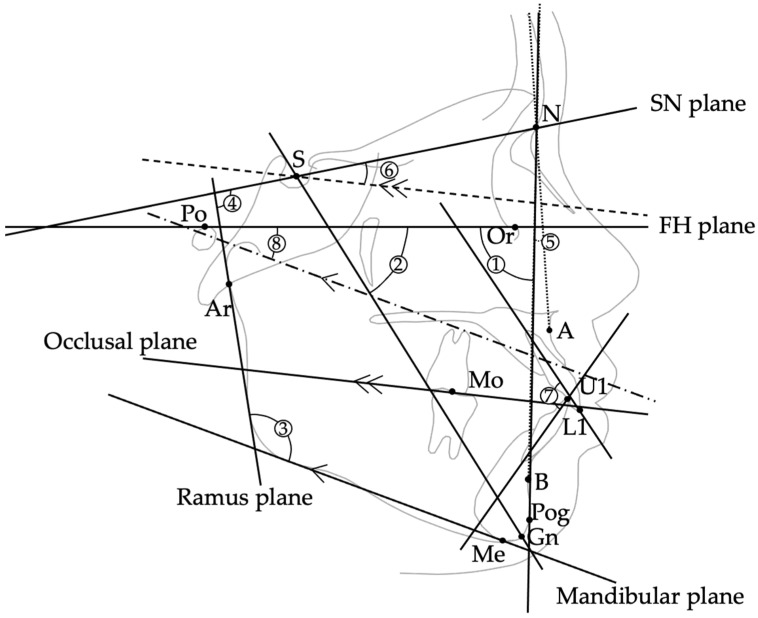
Schematic illustration showing the eight cephalometric angular measurements evaluated in this study. ① Facial angle: angle between the FH plane (Po–Or) and N–Pog line. ② *Y*-axis: angle between S–Gn line and FH plane. ③ Gonial angle: angle between the mandibular plane and ramus plane. ④ Ramus plane to SN: angle between the ramus plane and SN plane. ⑤ ANB: angle at Nasion formed by lines to Points A and B, representing sagittal maxillo-mandibular discrepancy. ⑥ Occlusal plane to SN: angle between the occlusal plane and SN plane. ⑦ Interincisal angle: angle between the long axes of the maxillary and mandibular central incisors. ⑧ FMA: angle between the mandibular plane and the FH plane.

Lateral cephalograms were obtained using a standardized head position with bilateral ear rods (CX-150ST 8000C, Asahi Roentgen Ind. Co., Ltd., Kyoto, Japan) under the following exposure settings: 150 kV, 250 mA, 0.32 s, with a focus-to-film distance of 1650 mm. These images were acquired by a certified radiologic technologist at the Department of Diagnostic Imaging, Tsurumi University Dental Hospital. Cephalometric tracings were digitized and analyzed using WinCeph ver. 11.0 (Rise Co., Ltd., Miyagi, Japan). The anatomical landmarks, reference planes, and angular measurements evaluated in this study are summarized in Table 1 and Figure 1.

The following 10 variables were assessed: ① Facial angle, ② *Y*-axis angle, ③ Gonial angle, ④ Ramus plane to SN, ⑤ ANB angle, ⑥ Occlusal plane to SN, ⑦ Interincisal angle, ⑧ FMA (Frankfort-mandibular plane angle), ⑨ Overjet, and ⑩ Overbite.

Overjet and overbite were measured on dental models using precision digital calipers.

All cephalometric measurements were performed by a single trained examiner (K.K., Department of Orthodontics, Tsurumi University School of Dental Medicine). To evaluate intra-examiner reliability, ten randomly selected radiographs were re-traced after a two-week interval, and the intra-class correlation coefficient (ICC) exceeded 0.95 for all cephalometric variables, indicating high repeatability. Measurement methodology and reliability assessment followed the protocol described by Xiong et al. (2020) [36].

### 2.4. Salivary Parameters

Stimulated whole saliva samples were evaluated using a multi-item saliva testing system (Salivary Multi Test^®^, LION Dental Products Co., Ltd., Tokyo, Japan), which enables chairside biochemical assessment of multiple salivary markers. This system (commercially developed from the AL-55 prototype) simultaneously quantifies seven salivary components, and in the present study, leukocyte count, protein concentration, and ammonia levels were analyzed as indicators of periodontal inflammatory activity [37,38,39].

Multi-item saliva testing was conducted according to the manufacturer’s protocol. Participants were instructed to refrain from eating, drinking, tooth brushing, and mouthwash use for at least 2 h prior to testing. After rinsing with 3 mL of distilled water for 10 s, a 10 μL sample of saliva was applied to Salivary Multi Test^®^ reagent strip. Colorimetric signals were analyzed using the multi-item saliva testing device one minute after application, and values for leukocytes, protein, and ammonia were recorded as relative reflectance values (0–100%).

Stimulated salivary flow rate was assessed using the Saxon test, wherein participants chewed sterile gauze for 2 min, and the increase in gauze weight was measured to calculate flow rate (g/2 min). This method is widely recognized as an objective clinical measure of salivary gland function [9].

In our department, salivary biochemical evaluation using a multi-item saliva testing system and stimulated salivary flow rate measurement (the Saxon test) are included in the standard orthodontic diagnostic protocol to assess oral hygiene conditions before, during, and after treatment, following ethics approval (Approval No. 1728). These assessments are performed only for patients who provide written informed consent, and the present study analyzed the values recorded at the initial examination.

### 2.5. Assessment of Mouth Breathing

Habitual mouth breathing was screened using six clinical indicators compiled from prior questionnaires and multidisciplinary diagnostic studies of habitual mouth breathing: (1) self-reported habitual mouth breathing, (2) lip incompetence at rest, (3) mouth open during sleep (self-/family report), (4) mentalis muscle strain on lip sealing, (5) buccal mucosal ridge (cheek line impression compatible with chronic lips-apart posture), and (6) history of nasal obstruction or allergic rhinitis. Importantly, participants with active nasal obstruction or symptomatic allergic rhinitis at the time of examination were excluded (see Section 2.1), whereas a past history of these conditions was included as an indicator of possible habitual mouth breathing. This distinction reflects the clinical consideration that mouth breathing may persist as a behavioral pattern even after airway patency improves [40], although such persistence cannot be confirmed definitively in a cross-sectional setting. These indicators reflect items repeatedly associated with mouth-breathing habit and/or reduced nasal patency in the literature, including questionnaire-based screening checklists and clinical-ENT composites [41,42]. In addition, nasal obstruction/allergic rhinitis is widely reported as a contributing condition for habitual oral breathing and dentofacial changes, supporting its inclusion as a screening indicator [29,43].

For the purposes of epidemiologic comparison and odds-ratio estimation, participants were classified dichotomously as mouth breathers when ≥1 of the six indicators were present (binary outcome: present/absent). This threshold prioritizes screening sensitivity in line with prior checklist approaches while acknowledging that multi-item scores have also been used; therefore, the present classification is interpreted as a screening criterion rather than a diagnostic gold standard [41,42].

### 2.6. Sample Size Calculation

A priori sample size estimation was performed using G*Power version 3.1.9.4 (Heinrich Heine University Düsseldorf, Düsseldorf, Germany). For the primary group comparison between the halitosis-positive and halitosis-negative groups, a two-tailed test was selected. The expected effect size was set at a medium effect size according to Cohen’s convention (d = 0.50) [44,45], with a significance level α = 0.05 and desired power 1 − β = 0.80. Under these conditions, the minimum required sample size per group was *n* = 64 (total *n* = 128). The final sample size in the present study (*n* = 234) therefore exceeded the required sample size for adequate statistical power.

For the multiple linear regression model, a priori considerations were also made. Based on Cohen’s convention for a medium effect size (f^2^ = 0.15), α = 0.05, 1 − β = 0.80, and 14 predictors, the minimum required sample size would be *n* = 135. In addition, according to Green’s rule of thumb for regression sample adequacy (*n* ≥ 50 + 8 m), the minimum recommended sample size for 14 predictors would be *n* = 162 [46]. The present sample size (*n* = 234) exceeded both criteria, indicating that the regression analysis was sufficiently powered at the design stage. The multiple linear regression model was prespecified with 14 predictors, and the corresponding a priori power analysis (f^2^ = 0.15; α = 0.05; 1 − β = 0.80) indicated an adequate sample size; therefore, no additional predictors were introduced post hoc.

### 2.7. Statistical Analysis

The distribution of continuous variables was examined using the Shapiro–Wilk test to assess normality. For comparisons between the halitosis-positive and halitosis-negative groups, independent *t*-tests were applied for normally distributed variables, whereas the Mann–Whitney U test was used for non-normally distributed variables, as appropriate. Additionally, exploratory sex-based comparisons of cephalometric and salivary parameters were performed using the same testing criteria to confirm the appropriateness of pooled analyses across males and females. To examine the association between craniofacial morphology, salivary environment, and halitosis severity, we performed a multiple linear regression with total VSC concentration (ppb) as the dependent variable. Independent variables were the ten cephalometric measurements (Facial angle, *Y*-axis, Gonial angle, Ramus plane to SN, ANB, Occlusal plane to SN, Interincisal angle, FMA, Overjet, Overbite) plus four salivary-related measures (stimulated salivary flow rate by the Saxon test, salivary leukocytes, salivary protein, and salivary ammonia), totaling 14 predictors. Cases with missing data were excluded listwise. Demographic covariates (age, sex) were not included to preserve the integrity of the prespecified model and the a priori power analysis. Regression outputs are presented as unstandardized coefficients (β) with 95% confidence intervals, along with R^2^ and adjusted R^2^.

Additionally, to assess the strength of association between mouth breathing (binary) and halitosis presence (total VSC ≥ 150 ppb), logistic regression analysis was conducted to calculate odds ratios (ORs) and 95% confidence intervals (CIs). All statistical analyses were performed using SPSS Statistics version 27.0 (IBM Japan, Tokyo, Japan). Statistical significance was set at *p* < 0.05.

## 3. Results

### 3.1. Normality Assessment and Sex-Based Exploratory Comparison

The Shapiro–Wilk test performed on the total sample (*n* = 234), indicated that ANB, overjet, overbite, salivary leukocyte count, salivary protein concentration, and stimulated salivary flow rate were not normally distributed (*p* < 0.05). In contrast, the remaining cephalometric and salivary variables demonstrated normal distributions (*p* ≥ 0.05). Accordingly, independent *t*-tests were used for normally distributed variables, whereas Mann–Whitney U tests were applied to non-normally distributed variables (Table S1).

In exploratory sex comparisons, total VSC concentration did not differ between males and females (median 76.00 [IQR 16.50–203.50] vs. 64.00 [16.50–234.00], *p* = 0.984). Several cephalometric variables showed sex-related differences, whereas salivary parameters were largely comparable except for higher salivary ammonia levels in males (44.48 ± 18.53 vs. 37.46 ± 17.51, *p* = 0.0067) (Table S2).

### 3.2. Comparison Between Halitosis-Positive and Halitosis-Negative Groups

Among the 234 participants, 79 (33.8%) were categorized in the halitosis-positive group (total VSC ≥ 150 ppb), and 155 (66.2%) were categorized in the halitosis-negative group. The total VSC concentration was considerably higher in the halitosis-positive group (mean 500.8 ± 421.7 ppb; median 372.0 ppb, IQR 233.0–589.0) compared with the halitosis-negative group (mean 42.1 ± 42.3 ppb; median 26.0 ppb, IQR 8.0–66.0).

The halitosis-positive group exhibited a significantly larger ANB angle (median 3.3° vs. 1.3°, *p* = 0.037), increased overjet (median 3.00 mm vs. 2.00 mm, *p* = 0.034), smaller interincisal angle (117.07° ± 14.85 vs. 122.25° ± 12.66, *p* = 0.009), and higher salivary ammonia levels (45.16 ± 18.84 vs. 36.93 ± 17.12, *p* = 0.001) compared with the halitosis-negative group (Table 2).

**Figure 2:**
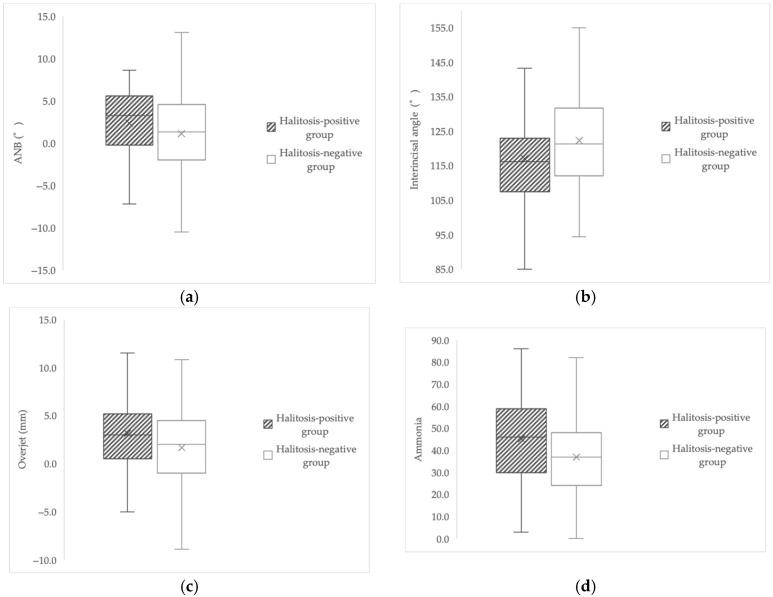
Boxplots illustrating the four variables that showed statistically significant differences between the halitosis-positive and halitosis-negative groups: (**a**) ANB angle (°), (**b**) overjet (mm), (**c**) interincisal angle (°), and (**d**) salivary ammonia levels. These boxplots are presented to visually demonstrate the group distributions observed in Table 2. In each boxplot, the left (hatched) box corresponds to the halitosis-positive group, and the right (solid) box corresponds to the halitosis-negative group. Consistent with the statistical results, the halitosis-positive group exhibited larger ANB angle and overjet, a smaller interincisal angle, and higher salivary ammonia levels compared with the halitosis-negative group.

No significant differences were observed between groups in facial angle, *Y*-axis angle, gonial angle, ramus inclination, occlusal plane inclination, FMA, salivary leukocyte count, salivary protein concentration, or stimulated salivary flow rate (g/2 min; all *p* > 0.05). These findings suggest that anteroposterior skeletal discrepancy, incisor inclination, and salivary ammonia-associated biochemical conditions are associated with the presence of halitosis. Figure 2 illustrates these group differences, demonstrating that the halitosis-positive group showed larger ANB, increased overjet, smaller interincisal angle, and higher salivary ammonia levels compared with the halitosis-negative group.

### 3.3. Multiple Linear Regression Analysis

Multiple linear regression analysis was performed with total VSC concentration (ppb) as the dependent variable and fourteen independent variables (ten cephalometric measurements and four salivary-related measures). Among these predictors, the interincisal angle was the only variable that remained significantly associated with total VSC levels (β = −4.57, *p* = 0.019) (Table 3). A smaller interincisal angle, indicating greater anterior incisor proclination, was associated with higher VSC concentration. The interincisal angle represents the relative inclination between the maxillary and mandibular incisors, and a reduced value reflects labial inclination of one or both incisors. None of the other cephalometric or salivary variables demonstrated significant independent associations in the multivariable model (all *p* ≥ 0.06). The overall model explained approximately 10.2% of the variance in VSC concentration (R^2^ = 0.102; adjusted R^2^ = 0.045; *n* = 234). These findings indicate that the interincisal angle was the only variable independently associated with total VSC levels.

### 3.4. Association Between Mouth Breathing and Halitosis

To examine the association between breathing pattern and halitosis, participants were classified according to the presence or absence of mouth breathing. The distribution was as follows: 53 individuals with mouth breathing and 26 without exhibited halitosis, whereas 47 individuals with mouth breathing and 108 without did not exhibit halitosis.

Logistic regression revealed that mouth breathing significantly increased the likelihood of halitosis (OR = 4.68, 95% CI: 2.62–8.38, *p* < 0.001), indicating that individuals exhibiting mouth breathing were approximately 4.7 times more likely to present clinically detectable halitosis than those with nasal breathing (Table 4).

This finding supports mouth breathing as an important functional factor related to halitosis in orthodontic patients.

## 4. Discussion

The present study examined the relationships among halitosis, craniofacial morphology, salivary biochemical characteristics, and mouth breathing in adult patients with malocclusion. To our knowledge, this is the first study to comprehensively evaluate structural, functional, and biochemical factors associated with oral malodor in adult patients with malocclusion. Previous studies have mainly focused on pediatric or post-treatment cohorts, whereas the present research provides novel cross-sectional evidence from untreated adult patients, integrating cephalometric morphology, salivary biomarkers, and functional breathing patterns. In addition, exploratory sex-based comparisons revealed no significant difference in total VSC concentration between males and females, supporting the validity of pooled analyses across sexes.

The results demonstrated that individuals with halitosis exhibited a larger ANB angle, increased overjet, a smaller interincisal angle, and higher salivary ammonia concentrations compared with those without halitosis. Furthermore, multiple regression analysis identified the interincisal angle as the only independent predictor of total VSC concentration, and logistic regression showed that mouth breathing was significantly associated with the presence of halitosis. These findings highlight the multidimensional nature of halitosis in adults with malocclusion, involving both morphological and functional factors.

A smaller interincisal angle, indicating greater anterior incisor proclination, was significantly associated with higher VSC levels in the present study. Consistent with previous reports, anterior dentoalveolar proclination—reflected by a reduced interincisal angle—has been associated with lip incompetence, increased exposure of the anterior oral cavity, reduced oral moisture retention, and an environment favorable for anaerobic bacterial growth and VSC production [17,18,19]. Notably, Koizumi et al. (2024) reported that individuals with a smaller interincisal angle exhibited compromised oral environment stability and a higher risk of caries, suggesting that anterior dentoalveolar morphology influences oral function beyond occlusal alignment alone [28]. Taken together, these findings support a mechanistic interpretation in which increased anterior incisor proclination may contribute to difficulty in maintaining lip closure at rest, fostering oral dryness and an environment conducive to anaerobic bacterial metabolism and VSC production. In addition to these morphological relationships, anterior incisor proclination may influence oral function through neuromuscular pathways. Increased proclination is frequently associated with difficulty maintaining passive lip seal, which can alter resting tongue posture and contribute to habitual mouth breathing, particularly during periods of low neuromuscular activity. Mouth breathing reduces intraoral humidity and accelerates evaporative water loss, resulting in thinning of the salivary film and diminished lubrication and buffering capacity. These physiological changes facilitate anaerobic bacterial metabolism and may increase VSC production. Thus, the interincisal angle may reflect a functional risk pathway influencing breathing mode and oral moisture stability, rather than acting as a direct structural determinant of halitosis.

Because the present study is cross-sectional, these associations cannot be interpreted as causal. The observed relationships may represent a directional tendency whereby craniofacial morphology influences breathing patterns, which subsequently modify salivary environmental conditions. Alternatively, they may be concurrent manifestations of broader neuromuscular or airway regulatory characteristics. Longitudinal and interventional studies, including evaluations before and after orthodontic treatment or airway therapy, are needed to clarify the temporal sequence of these interactions.

Higher salivary ammonia levels in the halitosis-positive group suggest increased bacterial proteolytic and ureolytic activity. Ammonia is generated when oral bacteria metabolize urea and amino acids, particularly on the dorsal surface of the tongue where anaerobic biofilms are dense [8,12,13]. Kozlovsky et al. demonstrated that ammonia concentration correlates strongly with the load of VSC-producing bacteria present in tongue coating [47], and Kazor et al. identified distinct microbial community shifts in patients with halitosis compared with controls, including enrichment of Fusobacterium, Prevotella, and Solobacterium species [48]. Therefore, the elevated ammonia concentrations observed in this study likely reflect not only variation in salivary biochemical composition but also broader microbial ecological dynamics. Although salivary ammonia levels were significantly higher in the halitosis-positive group, ammonia did not remain a significant independent predictor in the multivariable model. This pattern suggests that ammonia reflects secondary oral environmental changes (e.g., altered moisture dynamics associated with mouth breathing and anterior incisor proclination) rather than acting as a direct determinant of VSC production. These findings reinforce the importance of tongue coating management and salivary environment maintenance as key strategies for halitosis prevention.

Mouth breathing was associated with a 4.7-fold increased likelihood of clinically detectable halitosis. Mouth breathing can alter orofacial muscle balance and reduce salivary film stability, leading to dehydration of the oral mucosa and creating an environment favorable for the proliferation of anaerobic, VSC-producing bacteria [20,21,22,23,24,49,50,51]. Although mouth breathing is often initiated in childhood due to airway obstruction such as adenoidal hypertrophy or allergic rhinitis, previous reports indicate that it may persist into adulthood as a habitual breathing pattern influenced by impaired lip seal or neuromuscular adaptation [21,22,23,24]. Clinically, this suggests that assessment of breathing mode and lip closure competence should be incorporated into routine evaluation of halitosis, as structural correction of dental alignment alone may not sufficiently improve dryness-related malodor.

These findings underscore the importance of a multidimensional clinical approach to halitosis in adult patients with malocclusion. Craniofacial morphology—particularly anterior incisor inclination—may influence the ability to maintain an adequate lip seal, thereby supporting oral moisture retention and reducing the likelihood of an intraoral environment conducive to volatile sulfur compound production. In addition, elevated salivary ammonia levels suggest increased bacterial proteolytic activity, highlighting the relevance of assessing salivary biochemical conditions when evaluating malodor risk. The observed association between mouth breathing and halitosis further indicates that habitual breathing patterns and lip closure competence should be routinely considered during orthodontic examination and treatment planning. Morphological characteristics influencing tongue posture, lip seal, and self-cleaning ability may therefore modulate oral microbial ecology and predispose individuals to malodor even before orthodontic treatment [31,51]. Collectively, these perspectives align with contemporary international oral health frameworks that emphasize prevention-oriented and function-focused approaches to maintaining oral health [52].

Several limitations should be acknowledged. First, characteristics of the study population may influence the generalizability of the findings. The proportion of male and female participants was not balanced; although total VSC levels did not differ significantly between sexes (Table S2), this distribution may still limit generalizability. In addition, because the present study was conducted among adult patients seeking orthodontic treatment at a university dental hospital, skeletal morphology may not be representative of the general population. In particular, patients with skeletal Class II (i.e., larger ANB angle; maxillary protrusion tendency) or Class III (i.e., smaller ANB angle; mandibular protrusion tendency) relationships were likely overrepresented compared with individuals with skeletal Class I (ANB angle within the normal range). Therefore, caution is warranted when generalizing these findings to broader non-orthodontic populations. Second, the cross-sectional design precludes causal interpretation of the relationships identified in this study, and the temporal progression from craniofacial morphology or mouth breathing to halitosis cannot be determined. Longitudinal investigations assessing changes before and after orthodontic treatment or airway intervention would help clarify these directional associations. Third, mouth breathing classification relied on clinical evaluation and self-reported symptoms. Although this screening approach is widely used and clinically practical [44,45], incorporation of objective assessments such as rhinomanometry, acoustic rhinometry, or airflow monitoring would improve diagnostic precision. Furthermore, although individuals with active nasal obstruction or symptomatic allergic rhinitis were excluded, a past history of these conditions was included as a screening indicator of mouth-breathing tendency, because habitual mouth breathing may persist even after nasal airway patency is restored [40]. Therefore, the distinction between persistent habitual mouth breathing and breathing influenced by intermittent or residual airway obstruction could not be fully determined, representing an additional limitation of the present study. Moreover, the six clinical indicators used to classify mouth breathing were evaluated by multiple clinicians without formal inter-rater calibration. As a result, the classification may have prioritized sensitivity over specificity, potentially overestimating the prevalence of mouth breathing. Because of this variability, reclassification based on stricter criteria (e.g., ≥2 indicators) was not performed, as such an analysis would not yield reliable or interpretable results. Future studies should incorporate standardized examiner training and objective diagnostic measures to enhance reliability. Fourth, although multiple salivary biochemical parameters were evaluated, functional oral measurements such as tongue pressure, lip closing force, and masticatory performance were not assessed. A recent case–control study demonstrated that malocclusion severity is negatively associated with masticatory efficiency and that chewing function reflects the coordinated performance of the tongue and lip muscles [53], suggesting that soft-tissue functional capacity may also contribute to oral malodor development. Fifth, other potential confounding factors related to the oral environment were not assessed. In particular, tongue coating thickness, detailed oral hygiene status, and dietary intake of sulfur-containing foods were not evaluated, although they may influence anaerobic bacterial activity and VSC production independently of craniofacial morphology or mouth breathing. In addition, covariate-adjusted logistic regression could not be performed because individual-level scores for the mouth-breathing indicators were not available; therefore, the association between mouth breathing and halitosis is presented as a univariable model. Future studies incorporating quantitative coating indices, plaque assessments, dietary analysis, and standardized mouth-breathing metrics will help clarify the relative contributions of these factors.

Future research should therefore expand evaluation frameworks to include quantitative measures of tongue pressure and lip seal strength, as these may influence oral moisture retention and the microbial environment. In addition, assessment of urease activity could help clarify metabolic pathways contributing to ammonia production and its interaction with tongue-coating microbiota. Moreover, objective evaluation of airway patency and breathing mode would enhance diagnostic accuracy for mouth breathing. Integrating these functional and physiological assessments may allow the development of a more comprehensive model explaining halitosis risk in patients with malocclusion.

## 5. Conclusions

Halitosis in adult patients with malocclusion was associated with greater skeletal discrepancy (larger ANB), increased overjet, smaller interincisal angle, and elevated salivary ammonia levels. The interincisal angle emerged as the only independent cephalometric predictor of VSC concentration, and mouth breathing increased the likelihood of halitosis by approximately 4.7-fold. These findings support a multidimensional framework in which craniofacial morphology, salivary biochemical conditions, and breathing function collectively contribute to oral malodor.

## Figures and Tables

**Table 1:** Cephalometric landmarks, reference planes, and measurements (①–⑧: cephalometric measurements; ⑨–⑩: dental model measurements).

Item	Definition	Unit
(Landmarks/Points)		
S (Sella)	Center of the sella turcica	—
N (Nasion)	Most anterior point of the frontonasal suture	—
Ar (Articulare)	Point of intersection between the posterior border of the mandibular ramus and the cranial base (typically at the intersection of the posterior ramus contour and the basion–sella line)	—
Or (Orbitale)	Lowest point on the infraorbital rim	—
Po (Porion)	Highest point on the external auditory meatus	—
A-Point	Deepest point on the anterior contour of the maxilla	—
B-Point	Deepest point on the anterior contour of the mandible	—
Pog (Pogonion)	Point on the midsagittal outline of the mandibular symphysis that is tangent to a line perpendicular to the mandibular plane	—
Gn (Gnathion)	Point where the bisector of the angle formed by the N–Pog line and the mandibular plane intersects the mandibular symphysis in the midsagittal plane	—
Me (Menton)	Lowest point on the mandibular symphysis	—
U1/L1	Incisal edge of the maxillary/mandibular central incisor	—
Mo	Central point of upper/lower first molar occlusal contact	—
(Reference Planes)		
FH plane (Po–Or)	Plane connecting Porion and Orbitale	—
SN plane (S–N)	Plane connecting Sella and Nasion	—
Ramus plane	Tangent drawn from Articulare (Ar) along the posterior border of the mandibular ramus	—
Mandibular plane	Tangent drawn from Menton (Me) along the lower border of the mandible	—
Occlusal plane	Plane connecting midpoint of U1–L1 and Mo	—
(Cephalometric Measurements ①–⑧)		
① Facial angle	Angle between FH plane and N–Pog line	°
② *Y*-axis	Angle between S–Gn and FH plane	°
③ Gonial angle	Angle between mandibular plane and ramus plane	°
④ Ramus plane to SN	Angle between ramus plane and SN plane	°
⑤ ANB	Angle formed by A–N–B	°
⑥ Occlusal plane to SN	Angle between occlusal plane and SN plane	°
⑦ Interincisal angle	Angle between long axes of U1 and L1	°
⑧ FMA	Angle between mandibular plane and FH plane	°
(Dental Model Measurements ⑨–⑩)		
⑨ Overjet	Horizontal overlap of U1 over L1 (measured on dental models with a digital caliper)	mm
⑩ Overbite	Vertical overlap of U1 over L1 (measured on dental models with a digital caliper)	mm

**Table 2:** Comparison of craniofacial morphology and salivary parameters between the halitosis-positive and halitosis-negative groups.

Variable	Halitosis-Positive Group Mean/Median ± SD or (IQR)	Halitosis-Negative Group Mean/Median ± SD or (IQR)	*p*-Value
Facial angle (°)	86.96 ± 4.59	87.84 ± 4.78	0.17 ^a^
*Y*-axis (°)	63.98 ± 4.65	63.37 ± 4.83	0.28 ^a^
Gonial angle (°)	122.3 ± 6.1	121.9 ± 6.4	0.61 ^a^
Ramus plane to SN (°)	124.8 (122.0–128.4)	126.4 (122.7–129.7)	0.096 ^U^
ANB (°)	3.3 (−0.2–5.5)	1.3 (−2.0–4.6)	**0.037 ^U^**
Occlusal plane to SN (°)	16.7 ± 5.1	17.4 ± 5.3	0.42 ^a^
Interincisal angle (°)	117.07 ± 14.85	122.25 ± 12.66	**0.009 ^a^**
FMA (°)	27.3 ± 5.0	27.0 ± 4.9	0.64 ᵃ
Overjet (mm)	3.00 (0.50–5.10)	2.00 (−1.00–4.50)	**0.034 ^a^**
Overbite (mm)	2.00 (0.10–3.45)	2.50 (0.00–3.10)	0.27 ^U^
Salivary leukocytes	70.0 (51.5–82.0)	64.0 (46.5–78.0)	>0.05 ^U^
Salivary protein	47.0 (36.0–55.5)	43.0 (33.0–54.0)	>0.05 ^U^
Ammonia	45.16 ± 18.84	36.93 ± 17.12	**0.001 ^a^**
Stimulated salivary flow rate (g/2 min)	6.20 (4.30–7.20)	6.10 (4.85–7.30)	>0.05 ^U^

Values are expressed as mean ± standard deviation for normally distributed variables, and median (interquartile range) for non-normal variables. ^a^: independent *t*-test; ^U^: Mann–Whitney U test. Statistically significant differences are indicated in bold (*p* < 0.05). Halitosis was defined as total VSC ≥ 150 ppb measured by gas chromatography.

**Table 3:** Multiple linear regression analysis examining associations between cephalometric and salivary variables and total VSC concentration.

Predictor	β (Unstandardized)	*p*-Value	95% CI (Lower)	95% CI (Upper)	Interpretation
Interincisal angle (°)	−4.57	0.019	−8.37	−0.77	Smaller angle (greater proclination) → higher VSC (significant)
Gonial angle (°)	11.26	0.063	−0.61	23.13	Trend (not significant)
FMA (°)	−20.89	0.061	−42.77	0.99	Trend (not significant)
Overjet (mm)	12.44	0.085	−1.75	26.63	Trend (not significant)
Ramus plane to SN (°)	−6.85	0.194	−17.22	3.51	Not significant
ANB (°)	10.69	0.225	−6.62	28.00	Not significant
Facial angle (°)	12.59	0.390	−16.21	41.40	Not significant
*Y*-axis (°)	15.93	0.340	−16.88	48.74	Not significant
Occlusal plane to SN (°)	4.43	0.469	−7.59	16.44	Not significant
Overbite (mm)	3.13	0.741	−15.50	21.76	Not significant
Stimulated salivary flow rate (Saxon)	2.89	0.776	−17.06	22.84	Not significant
Salivary leukocytes	0.46	0.647	−1.51	2.42	Not significant
Salivary protein	−0.56	0.717	−3.60	2.48	Not significant
Salivary ammonia	0.75	0.577	−1.90	3.41	Not significant

The dependent variable was total VSC concentration (ppb). Independent variables included ten cephalometric measurements (Facial angle, *Y*-axis, Gonial angle, Ramus plane to SN, ANB, Occlusal plane to SN, Interincisal angle, FMA, Overjet, and Overbite) and four salivary-related measures (stimulated salivary flow rate, salivary leukocytes, salivary protein, and salivary ammonia). Regression coefficients (β) are unstandardized. A smaller interincisal angle reflects greater anterior incisor proclination. Statistical significance was set at *p* < 0.05. Variables in Table 3 are presented in order of their statistical contribution to the model, with the significant predictor listed first.

**Table 4:** Logistic regression analysis assessing the association between mouth breathing and the presence of halitosis.

Variable	Halitosis (+)	Halitosis (−)	OR	95% CI	*p*-Value
Mouth breathing present	53	47	4.68	2.62–8.38	<0.001
Mouth breathing absent	26	108	Reference	—	—

Halitosis was defined as total VSC ≥ 150 ppb. Mouth breathing was classified based on the clinical screening criteria described in Section 2.5. Odds ratio (OR) and 95% confidence intervals (CI) were calculated using binomial logistic regression. Statistical significance was set at *p* < 0.05.

## Data Availability

All clinical data are available in the Supplementary Materials.

## Supplementary Materials

The following supporting information can be downloaded at: https://www.mdpi.com/article/10.3390/jcm14238293/s1, Figure S1: Conceptual framework of the hypothesized pathways linking craniofacial morphology, mouth breathing, salivary environment, and VSC accumulation leading to halitosis. Table S1: Shapiro–Wilk Test for Normality (Total Sample, *n* = 234), Table S2: Sex-based comparisons of Total VSC, cephalometric, and salivary variables, File S1: All the data analyzed in this study.
