# Supplementary material for: Associations Between Halitosis and Craniofacial Morphology, Salivary Biochemical Parameters, and Mouth Breathing in Adult Patients with Malocclusion: A Cross-Sectional Study
Source: J Clin Med. 2025 Nov 22;14(23):8293. doi: 10.3390/jcm14238293 (PMC12692599; doi:10.3390/jcm14238293)
Supplement: Supplementary file 1 [file jcm-14-08293-s001.zip › Supplemental materials Revise/Figure S1, Table S1,S2.pptx]

## Slide 1
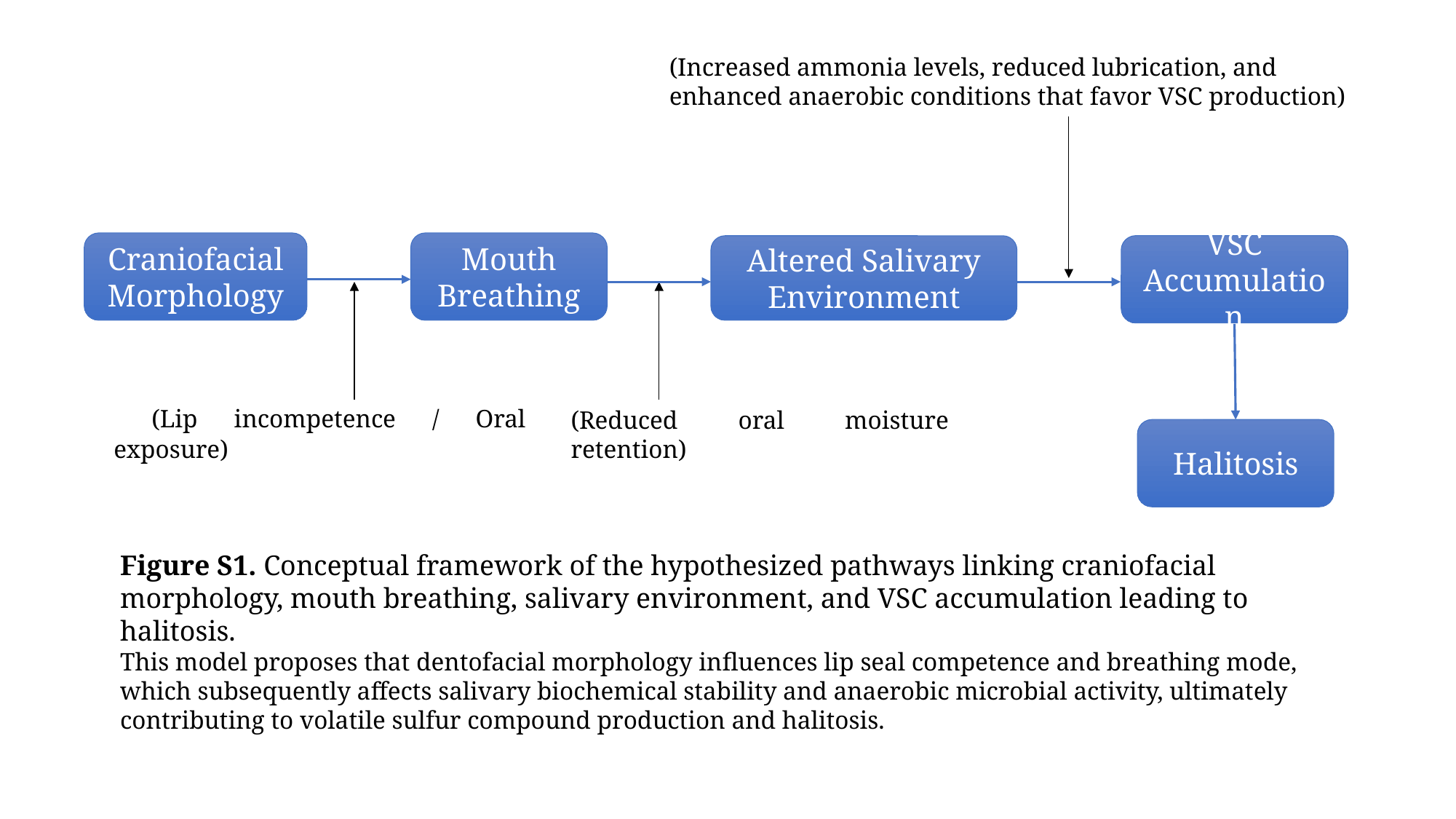

(Increased ammonia levels, reduced lubrication, and enhanced anaerobic conditions that favor VSC production)
Craniofacial
Morphology
Mouth
Breathing
VSC
Accumulation
Altered Salivary
Environment
 (Lip incompetence / Oral exposure)
(Reduced oral moisture retention)
Halitosis
Figure S1. Conceptual framework of the hypothesized pathways linking craniofacial morphology, mouth breathing, salivary environment, and VSC accumulation leading to halitosis.
This model proposes that dentofacial morphology influences lip seal competence and breathing mode, which subsequently affects salivary biochemical stability and anaerobic microbial activity, ultimately contributing to volatile sulfur compound production and halitosis.

## Slide 2
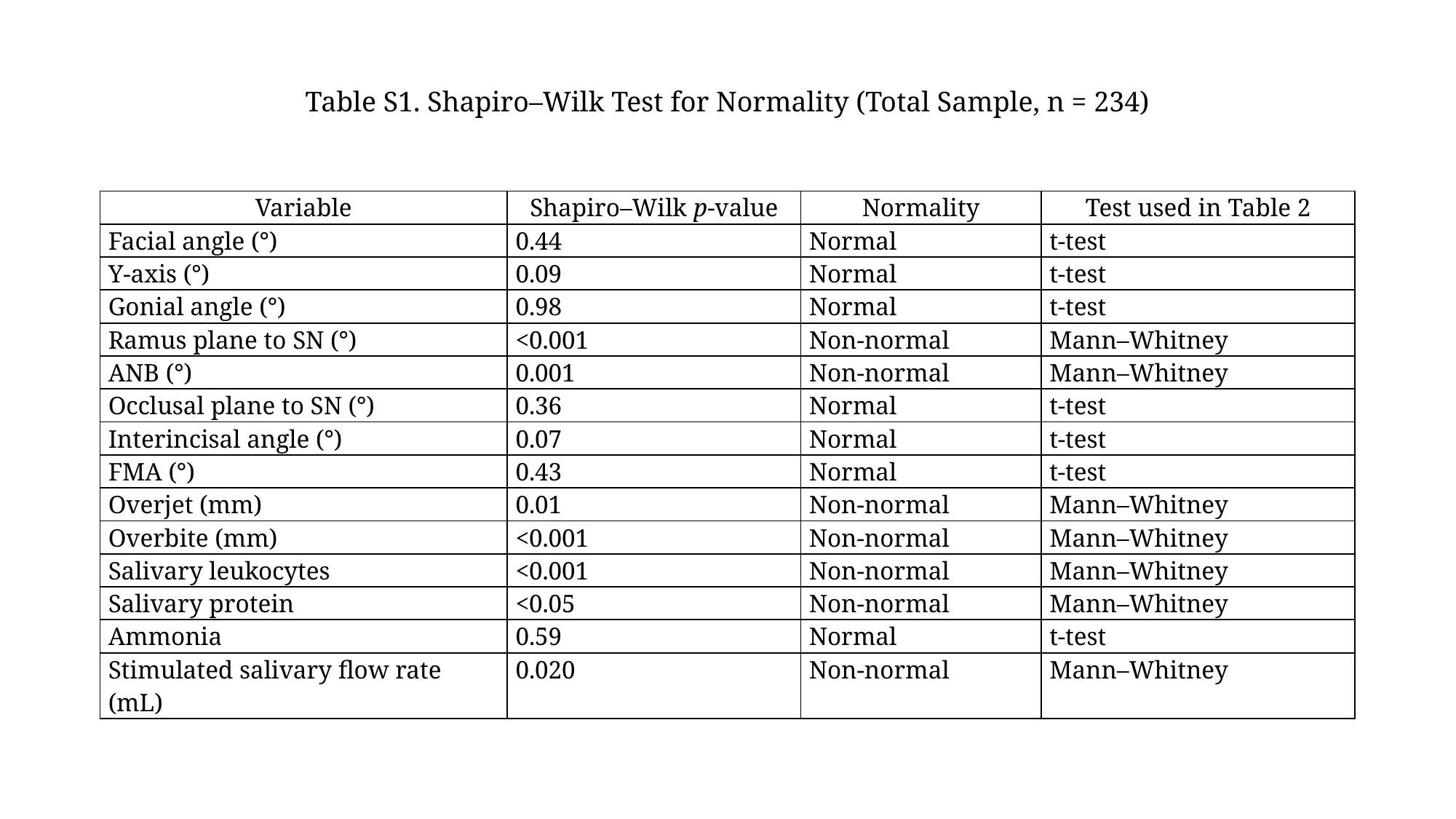

Table S1. Shapiro–Wilk Test for Normality (Total Sample, n = 234)
| Variable | Shapiro–Wilk p-value | Normality | Test used in Table 2 |
| --- | --- | --- | --- |
| Facial angle (°) | 0.44 | Normal | t-test |
| Y-axis (°) | 0.09 | Normal | t-test |
| Gonial angle (°) | 0.98 | Normal | t-test |
| Ramus plane to SN (°) | <0.001 | Non-normal | Mann–Whitney |
| ANB (°) | 0.001 | Non-normal | Mann–Whitney |
| Occlusal plane to SN (°) | 0.36 | Normal | t-test |
| Interincisal angle (°) | 0.07 | Normal | t-test |
| FMA (°) | 0.43 | Normal | t-test |
| Overjet (mm) | 0.01 | Non-normal | Mann–Whitney |
| Overbite (mm) | <0.001 | Non-normal | Mann–Whitney |
| Salivary leukocytes | <0.001 | Non-normal | Mann–Whitney |
| Salivary protein | <0.05 | Non-normal | Mann–Whitney |
| Ammonia | 0.59 | Normal | t-test |
| Stimulated salivary flow rate (mL) | 0.020 | Non-normal | Mann–Whitney |

## Slide 3
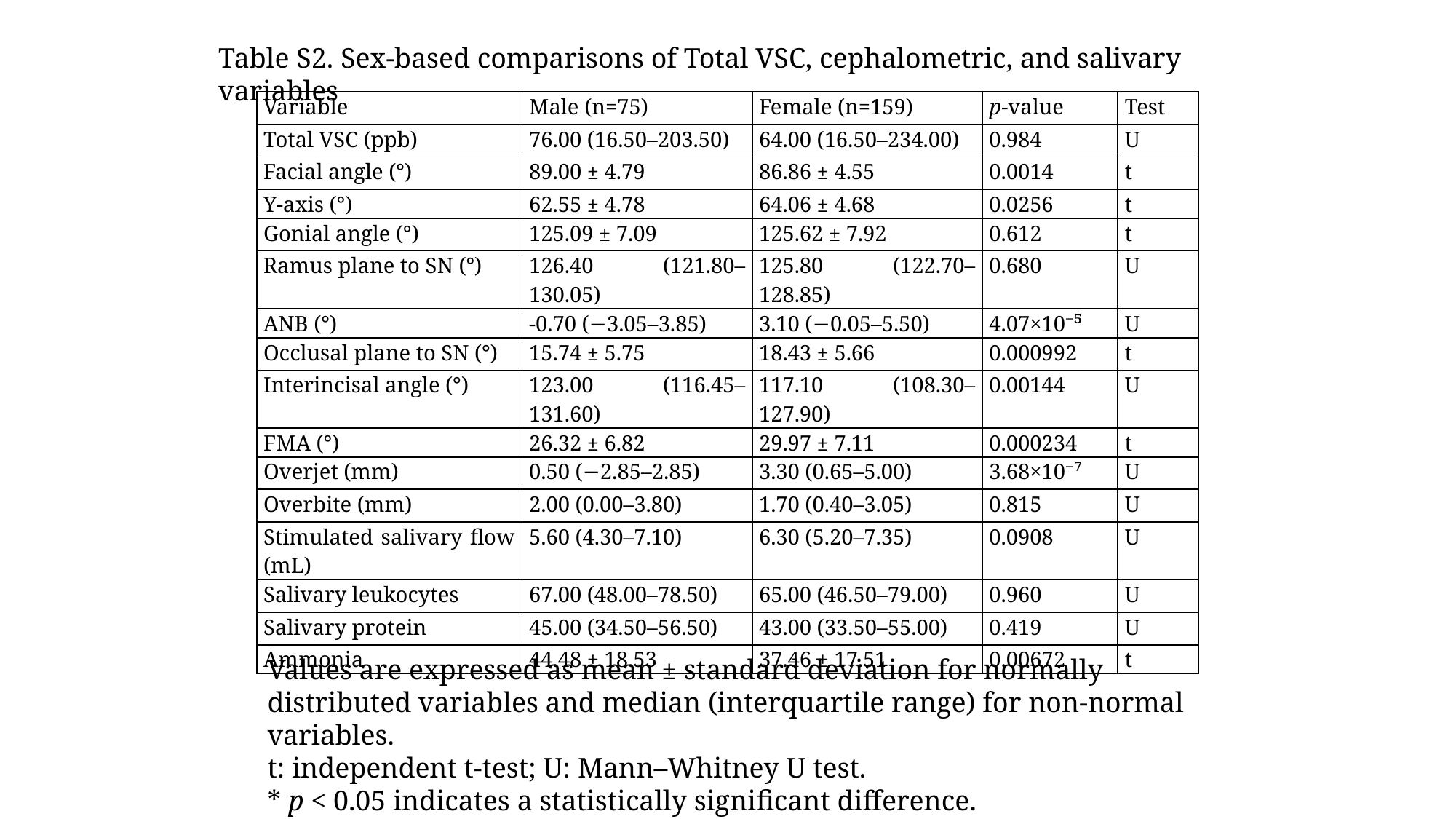

Table S2. Sex-based comparisons of Total VSC, cephalometric, and salivary variables
| Variable | Male (n=75) | Female (n=159) | p-value | Test |
| --- | --- | --- | --- | --- |
| Total VSC (ppb) | 76.00 (16.50–203.50) | 64.00 (16.50–234.00) | 0.984 | U |
| Facial angle (°) | 89.00 ± 4.79 | 86.86 ± 4.55 | 0.0014 | t |
| Y-axis (°) | 62.55 ± 4.78 | 64.06 ± 4.68 | 0.0256 | t |
| Gonial angle (°) | 125.09 ± 7.09 | 125.62 ± 7.92 | 0.612 | t |
| Ramus plane to SN (°) | 126.40 (121.80–130.05) | 125.80 (122.70–128.85) | 0.680 | U |
| ANB (°) | -0.70 (−3.05–3.85) | 3.10 (−0.05–5.50) | 4.07×10⁻⁵ | U |
| Occlusal plane to SN (°) | 15.74 ± 5.75 | 18.43 ± 5.66 | 0.000992 | t |
| Interincisal angle (°) | 123.00 (116.45–131.60) | 117.10 (108.30–127.90) | 0.00144 | U |
| FMA (°) | 26.32 ± 6.82 | 29.97 ± 7.11 | 0.000234 | t |
| Overjet (mm) | 0.50 (−2.85–2.85) | 3.30 (0.65–5.00) | 3.68×10⁻⁷ | U |
| Overbite (mm) | 2.00 (0.00–3.80) | 1.70 (0.40–3.05) | 0.815 | U |
| Stimulated salivary flow (mL) | 5.60 (4.30–7.10) | 6.30 (5.20–7.35) | 0.0908 | U |
| Salivary leukocytes | 67.00 (48.00–78.50) | 65.00 (46.50–79.00) | 0.960 | U |
| Salivary protein | 45.00 (34.50–56.50) | 43.00 (33.50–55.00) | 0.419 | U |
| Ammonia | 44.48 ± 18.53 | 37.46 ± 17.51 | 0.00672 | t |
Values are expressed as mean ± standard deviation for normally distributed variables and median (interquartile range) for non-normal variables.
t: independent t-test; U: Mann–Whitney U test.
* p < 0.05 indicates a statistically significant difference.
